# Pyroptosis: Novel Targets in Molecular Mechanisms and Drug Therapy Research for Myocardial Ischemia Reperfusion Injury

**DOI:** 10.31083/RCM48469

**Published:** 2026-05-21

**Authors:** Dong Zhang, Hui Wu, ShengYu Gong, Gang Zhou, Qing Zhang, Song Li, Wei Yang

**Affiliations:** ^1^Interventional Diagnosis and Treatment Center, Yichang Central People's Hospital, 443000 Yichang, Hubei, China; ^2^Institute of Cardiovascular Disease, China Three Gorges University, 443005 Yichang, Hubei, China; ^3^Department of Cardiology, Yichang Central People's Hospital, 443000 Yichang, Hubei, China; ^4^Central Laboratory, The First College of Clinical Medical Science, China Three Gorges University, 443005 Yichang, Hubei, China; ^5^Pharmaceutical Department, Yichang Central People's Hospital, 443000 Yichang, Hubei, China

**Keywords:** pyroptosis, acute myocardial infarction, myocardial ischemia reperfusion injury, NOD-like receptor protein 3, reactive oxygen species

## Abstract

Myocardial ischemia-reperfusion injury is a significant complication of reperfusion therapy and a primary cause of mortality in patients with acute myocardial infarction. The pathogenic mechanism involved in myocardial ischemia-reperfusion injury is intricate, and effective preventive and therapeutic strategies remain limited in clinical practice. Recently, pyroptosis has emerged as a novel regulatory form of cell death and has attracted widespread attention as a key focus in the study of disease mechanisms and therapeutic targets. Studies indicate a close association between pyroptosis and the pathophysiological processes underlying myocardial ischemia-reperfusion injury. This article provides a comprehensive review of recent advances in research on pyroptosis in the context of myocardial ischemia-reperfusion injury. Therefore, this review aims to offer new insights into the prevention and treatment of myocardial ischemia-reperfusion injury while minimizing redundancy in the existing literature.

## 1. Introduction

Acute myocardial infarction (AMI) is a severe cardiovascular event typically caused by the sudden occlusion of a coronary artery, usually due to thrombus formation, rupture of an atherosclerotic plaque, or other factors [1]. Abrupt arterial blockage restricts blood flow to the myocardium, leading to cellular damage from oxygen and nutrient deprivation. Even with prompt treatment, myocardial infarction can result in cardiomyocyte necrosis. Following myocardial necrosis, cardiac function may be impaired, potentially leading to serious complications such as heart failure [2]. Common treatments for AMI include thrombolytic medications, percutaneous coronary intervention, and coronary artery bypass grafting. Restoration of coronary blood flow during reperfusion reintroduces oxygen and nutrients to the ischemic myocardium [3]. However, this process may also cause additional damage, known as myocardial ischemia-reperfusion injury (MIRI) [4].

Pyroptosis, identified in recent years, is a distinct form of cell death that differs from both apoptosis and necrosis [5]. As an inflammatory form of cell death, pyroptosis is closely associated with immune activation and the release of inflammatory mediators, playing a crucial role in combating infectious pathogens and cellular damage [6]. Pyroptosis typically involves the formation and activation of inflammasomes, which are intracellular multiprotein complexes. Upon sensing infection- or damage-associated signals, inflammasomes are activated, triggering pyroptosis [7]. This process promotes the release of inflammatory mediators into the surrounding environment, thereby activating immune cells and inflammatory responses, aiding in defense against infections. However, under certain conditions, pyroptosis can also cause excessive inflammation and tissue damage [8]. While pyroptosis plays a vital role in immune responses and inflammation, the dysregulation or overactivation of this process can have adverse effects on health and is associated with various diseases, including cardiovascular diseases [9], infectious diseases [10], and autoimmune disorders [11].

Recently, pyroptosis has been shown to be closely associated with MIRI [12]. However, the precise mechanisms underlying MIRI remain incompletely understood. Thus, this review primarily focuses on the relationship between MIRI and pyroptosis-associated signaling pathways, aiming to provide a theoretical basis for future research and the development of novel clinical therapeutic targets.

## 2. Pyroptosis and MIRI 

### 2.1 Oxidative Stress 

During myocardial ischemia-reperfusion, insufficient oxygen and nutrient supply disrupt cardiomyocyte homeostasis, leading to an imbalance in the intracellular redox state [13]. Upon reperfusion, there is an increased generation of intracellular reactive oxygen species (ROS), including superoxide radicals, hydrogen peroxide, and hydroxyl radicals [14, 15]. Under normal circumstances, moderate ROS levels help activate inflammatory signaling pathways and promote the synthesis and secretion of inflammatory mediators, thereby supporting a normal inflammatory response [16]. However, during myocardial ischemia-reperfusion, excessive ROS (*e*.*g*., superoxide) not only cause direct cellular damage but also activate the NLR family pyrin domain-containing 3 (NLRP3) inflammasome via several molecular intermediates [17]. For example, ROS promote the dissociation of thioredoxin-interacting protein (TXNIP) from thioredoxin, enabling TXNIP to bind and activate NLRP3 [18]. Additionally, ROS can induce mitochondrial dysfunction, leading to the release of mitochondrial DNA and cardiolipin, which further activate NLRP3 inflammasome assembly [19]. These events facilitate the recruitment of apoptosis-associated speck-like protein containing a CARD (ASC) and pro-caspase-1, resulting in caspase-1 activation, gasdermin D (GSDMD) cleavage, and pyroptotic cell death, thereby exacerbating MIRI [13, 20]. Furthermore, oxidative stress can cause membrane damage, impair mitochondrial function, and ultimately lead to cardiomyocyte and tissue injury or even death [21]. Studies have found that, in a myocardial infarction model, inducing ROS activation with nitrogen oxide (NOx), followed by ROS overexpression, can promote the formation of inflammasomes composed of caspase-1/NLRP3/ASC, thereby inducing pyroptosis in cardiomyocytes and worsening reperfusion injury [22]. Consistently, in a mouse model of myocardial infarction, geniposide was reported to prevent cleavage of the key pyroptosis protein GSDMD, thereby inhibiting pyroptosis and improving cardiac function [23]. Collectively, these studies suggest that excessive ROS can induce pyroptosis in cardiomyocytes and exacerbate MIRI.

### 2.2 Calcium Ion Overload

Early reports have suggested a close relationship between calcium overload and pyroptosis [24]. During myocardial ischemia, the function of cell membrane channels is impaired, leading to the accumulation of intracellular calcium ions (Ca^2+^). Upon reperfusion, a significant influx of Ca^2+^ into cells triggers a series of adverse biochemical reactions, including the activation of oxidative stress responses and inflammation, ultimately leading to cell death [25]. Studies have shown that Ca^2+^ overload can also impair mitochondrial function, induce mitochondrial permeability transition, and promote the release of pro-death signaling molecules stored within mitochondria. These signaling molecules, in turn, can activate pyroptotic pathways, ultimately resulting in MIRI [26]. Notably, some research teams have treated rat cardiomyocytes with hydrogen peroxide (H_2_O_2_) and observed increased Ca^2+^ levels and excessive ROS production, which eventually damage the cell membrane. Following membrane disruption, cardiomyocytes release significant amounts of cytokines, interferons, chemokines, and other factors, thereby inducing pyroptosis and exacerbating myocardial injury [27]. In summary, there is substantial evidence to infer that Ca^2+^ overload is closely associated with pyroptosis and can exacerbate MIRI.

### 2.3 Endothelial Cell Injury

In myocardial ischemia, reduced blood flow and inadequate oxygen and nutrient supply not only damage cardiomyocytes but also affect endothelial cells in the surrounding blood vessels [21]. When coronary arteries are reperfused, the rapid restoration of oxygen and nutrient supply to the myocardium can paradoxically exacerbate endothelial cell damage. During reperfusion, cells may undergo pathological processes, including the generation of oxygen-free radicals, inflammatory responses, and pyroptosis [28, 29]. It has been found that inhibiting microvascular endothelial cell damage and pyroptosis in MIRI mouse models can alleviate myocardial ischemia/reperfusion injury and protect cardiovascular microvascular function [30]. Furthermore, in a foundational study using a reperfusion injury model, endothelial injury was shown to be mediated by regulating caspase family activation through downregulation of the Beclin 1 (*BECN1*) gene [31]. Based on these findings, we infer that pyroptosis may disrupt endothelial cell function and thereby contribute to the regulation of MIRI.

### 2.4 Adenine Nucleoside Triphosphate Imbalance

It is well known that mitochondria are the cellular powerhouses responsible for energy production, and the stability of mitochondrial function is crucial for the normal physiological activities of cardiac cells [32]. During MIRI, mitochondrial dysfunction occurs, often leading to impaired adenosine triphosphate (ATP) synthesis. ATP is a key energy molecule essential for maintaining cell survival and function [33]. Upon restoration of blood flow to the infarcted area, a significant and instantaneous replenishment of ATP occurs, a major cause of ATP disruption during ischemia [33]. Normal ATP levels help maintain intracellular potassium ion (K^+^) concentrations, thereby preventing GSDMD activation and membrane rupture, ultimately reducing pyroptosis [34]. Once ATP is released into the extracellular space, the molecule can act as a danger signal that triggers inflammasome activation [35]. The inflammasome is a multiprotein complex, including the NLRP3 inflammasome, which can activate the production of the proinflammatory cytokines interleukin-1β (IL-1β) and interleukin-18 (IL-18) [36]. One study found that ATP disruption, increased ROS production [37], inflammasome formation, and recruitment of the classical pyroptosis-related protein caspase-1 collectively promote pyroptosis through GSDMD activation [38]. In conclusion, ATP disruption can induce pyroptosis, thereby regulating MIRI.

**Fig. 1.**
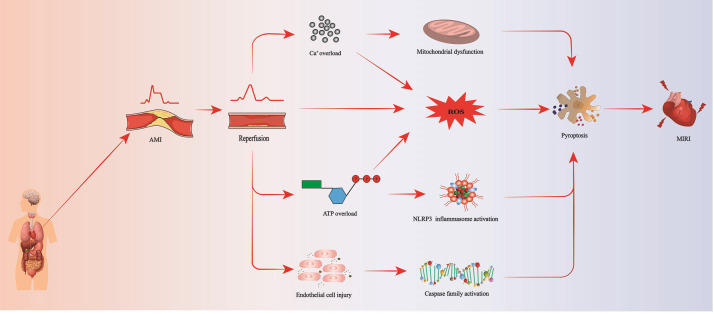
**Pyroptosis pathway leading to MIRI**. AMI, acute myocardial infarction; ROS, reactive oxygen species; ATP, adenosine triphosphate; NLRP3, NLR family pyrin domain-containing 3; MIRI, myocardial ischemia reperfusion injury.

In summary, pyroptosis in MIRI is not an independent process but is influenced by multiple interrelated factors (Fig. 1).

## 3. Morphological and Molecular Characteristics of Pyroptosis

### 3.1 Morphology of Pyroptosis 

Pyroptosis exhibits specific morphological characteristics distinct from other forms of cell death, such as apoptosis, autophagy, and necrosis. During pyroptosis, damaged cells undergo pronounced cytoplasmic swelling, leading to a significant increase in cell volume, attributed to abnormal accumulation of intracellular water and ions [39]. Meanwhile, pyroptosis is typically associated with cell membrane rupture, primarily due to the formation of pores or channels. This differs from the release of cytoplasmic bodies during apoptosis. Additionally, pyroptosis induces DNA fragmentation, producing characteristic DNA fragments that are distinct from those produced by the gradual degradation of DNA in apoptosis [40]. These pores are often mediated by GSDMD, which is cleaved to produce the membrane-pore-forming GSDMD-N. This fragment, in turn, forms pores on the cell membrane, leading to membrane disruption and the release of intracellular contents into the extracellular space. This controlled pore formation is a crucial distinction between pyroptosis and necrosis, in which plasma membrane rupture is uncontrolled [41, 42]. Pyroptosis is also distinct from autophagy, where double-membraned autophagosomes encapsulate cytoplasmic materials and then fuse with lysosomes for content digestion [43]. Moreover, pyroptosis is an inflammatory form of cell death, typically accompanied by inflammasome activation. Inflammasomes are multiprotein complexes activated upon sensing infection or cellular damage signals. Additionally, inflammasomes trigger an inflammatory response, leading to the release of proinflammatory cytokines such as IL-1β and IL-18. These cytokines participate in triggering immune responses and act as the final effectors of pyroptosis [44]. Owing to these distinctive morphological features, pyroptosis exerts unique effects on the pathophysiological processes of organisms (Table 1).

**Table 1.** **Comparison of different cell death modes**.

Classification	Key stimuli	Key proteins	Morphological changes
Pyroptosis	LPS, ATP, ROS, bacterial infection	Caspase-1/4/11, IL-18, GSDMD, IL-1β,	Cell swelling, plasma membrane pore formation, release of cellular contents, intact nucleus with DNA fragmentation
Apoptosis	DNA damage, growth factor withdrawal, TNF	Caspase-3/8/9, p53, Bcl-2 family	Cell shrinkage, chromatin condensation, nuclear fragmentation, formation of apoptotic bodies, intact plasma membrane
Autophagy	Nutrient starvation, ER stress, rapamycin	ATG5, ATG7, beclin-1, LC3, p62	Formation of double-membraned autophagosomes, cytoplasmic vacuolization, degradation of organelles, partial chromatin condensation
Necroptosis	TNF, LPS, viral infection	RIPK1, RIPK3, MLKL	Organelle swelling, plasma membrane rupture, moderate chromatin condensation, release of DAMPs
Necrosis	Physical/chemical trauma, abrupt ATP depletion	Nonspecific (accidental)	Rapid cell swelling, loss of membrane integrity, organelle disintegration, random DNA degradation, no typical apoptotic or autophagic features

GSDMD, gasdermin D; IL, interleukin; DAMPs, damage-associated molecular patterns; LPS, lipopolysaccharide; TNF, tumor necrosis factor; ER, endoplasmic reticulum; ATG, autophagy related; LC, microtubule-associated protein 1 light; RIPK, receptor-interacting protein kinase; MLKL, mixed lineage kinase domain-like protein.

### 3.2 Molecular Characteristics of Pyroptosis

Pyroptosis is a distinct form of cell death with unique molecular characteristics that can be used to distinguish this process from other modes of cell death. The following are the key molecular features of pyroptosis.

#### 3.2.1 Caspase Family

Caspases constitute a conserved family of cysteine proteases, with a structure comprising an N-terminal caspase recruitment domain, a central large catalytic domain, and a C-terminal small catalytic subunit domain. Together, these domains collaborate to form the enzymatic active site [45]. Functionally, caspases can be categorized based on the associated role and activity, including those associated with inflammation, such as caspase-1/3/4/5/6/7/8/11 and 12 [46], which mediate pyroptosis through distinct structures and activities [47]. Caspases related to apoptosis include caspase-2, 8, 9, and 10 [48]. Meanwhile, caspase-8, which participates in both pyroptosis and apoptosis, can activate NLRP3 and cleave GSDMD [49]. One study found that caspase-1, while binding to the N- and C-terminal connectors of GSDMD, also interacts with the hydrophobic pocket at the distant end of the N-terminal domain through anti-parallel β-sheets on the L2 and L2’ loops. This suggests that caspase-1 can mediate the specific cleavage of GSDMD via multiple mechanisms [50]. Overall, the caspase family plays a central role in regulating pyroptosis.

#### 3.2.2 Inflammasomes

The formation of inflammasomes is a critical feature of pyroptosis, in which, upon sensing danger signals inside and outside the cell, inflammasomes assemble and activate, triggering inflammatory responses and pyroptosis [51]. Pyroptosis is typically triggered by specific stimuli, such as those from infectious pathogens, injury, or other danger signals. These signals are recognized by intracellular sensors, primarily members of the pattern recognition receptor (PRRs) family, including nucleotide-binding oligomerization domain (NOD) and leucine-rich repeat (LRR) proteins, such as NOD-like receptor (NLR) family members, the absent in melanoma 2 (AIM2) receptor, and the tripartite motif (TRIM) family protein pyrin, among which NLRP1, NLRP3, NLR family CARD domain containing 4 (NLRC4), and the mouse proteins Nlrp1a and Nlrp1b are confirmed to form inflammasomes [52]. Once inflammatory signals are sensed, NLRP3 and ASC, along with caspase-1, assemble to form the inflammasome complex [53]. Pyroptosis can contribute to ox-LDL-induced macrophage death, promoting the formation of late necrotic cores in atherosclerotic plaques and increasing the instability of fibrous plaques by activating NLRP3 inflammasomes [54]. Studies further suggest that pyroptosis in endothelial cells, vascular smooth muscle cells, and macrophages, among others, contributes to the formation and progression of atherosclerosis by inducing NLRP3 inflammasome formation and regulating pyroptotic cell death [55]. Collectively, these findings highlight the crucial role of inflammasomes in the initiation and execution of pyroptosis.

#### 3.2.3 GSDMD

GSDMD is a crucial molecule in pyroptosis, comprising highly conserved N- and C-terminal functional domains. The N-terminal domain typically targets the cell membrane, leading to cell swelling, rupture, and the release of intracellular inflammatory factors [56]. In the resting state, the C-terminal and N-terminal are in an autoinhibitory state, preventing pyroptosis and maintaining cellular homeostasis [57]. During pyroptosis, damage-associated molecular patterns (DAMPs) released by damaged cells are recognized by inflammasomes in immune cells, thereby activating the classical pyroptotic pathway through caspase-1. Once activated, caspase-1 selectively cleaves GSDMD, generating the N-terminal GSDMD fragment, a pore-forming peptide. This peptide binds to phospholipids in the cell membrane, causing swelling and rupture of the immune cell membrane, thereby promoting the release of inflammatory factors such as IL-1β and IL-18 [58]. Research has found that GSDMD, identified through genomic sequencing as a downstream target of caspase-4/11, can be directly recognized and cleaved, thereby triggering pyroptosis, leading to cell membrane rupture and the release of intracellular contents, which constitute one of the morphological features of pyroptosis [58]. Meanwhile, studies have confirmed that GSDMD is a key substrate for the activation of caspase-1, -4, -5, and -11, serving as the primary effector mediating pyroptosis [59]. Collectively, these findings underscore the significance of GSDMD as the executor of pyroptosis.

**Fig. 2.**
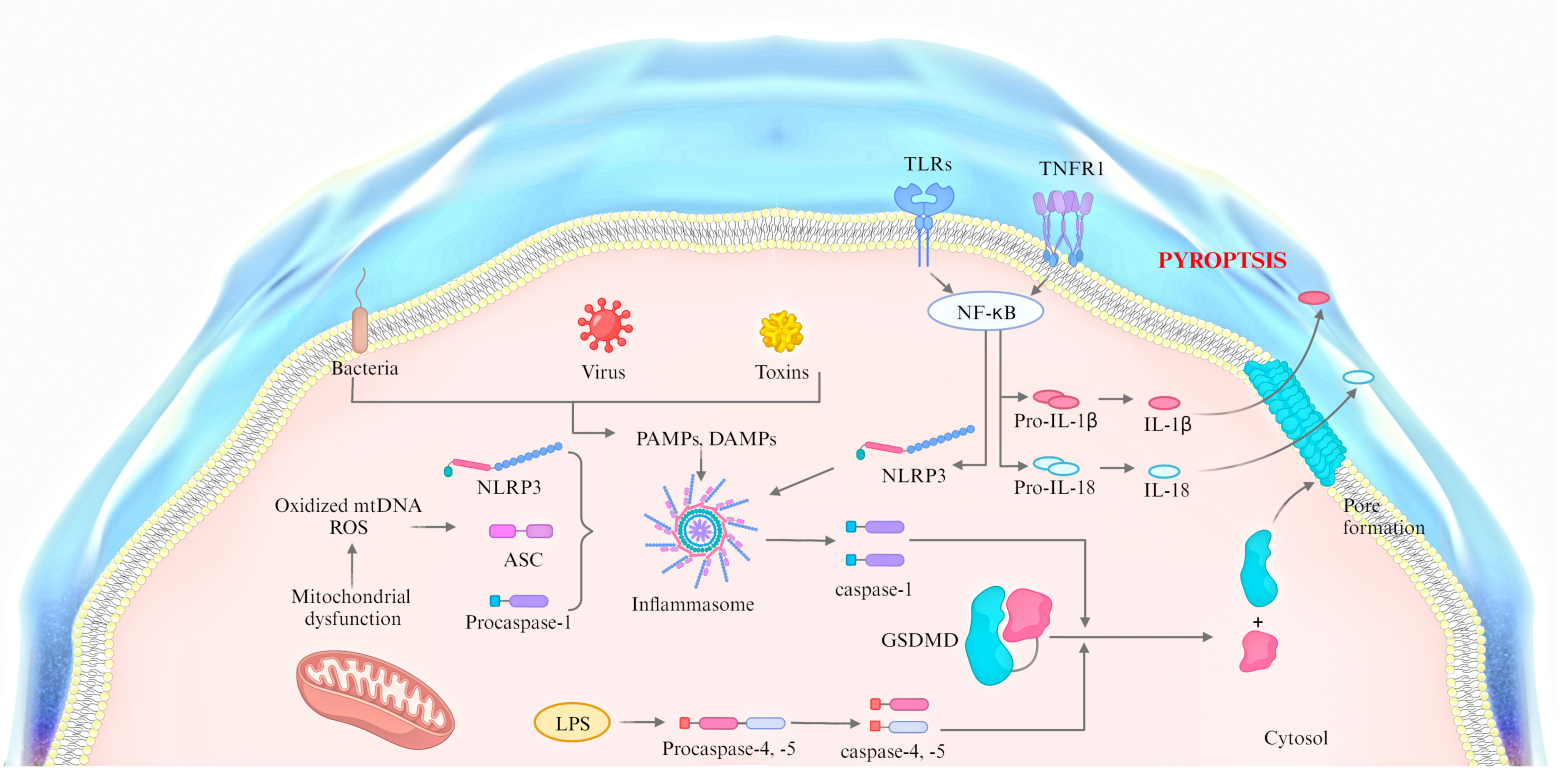
**The mechanisms of pyroptosis**. ASC, apoptosis-associated speck-like protein containing a CARD; TLRs, toll-like receptors; TNFR, tumor necrosis factor receptor; PAMPs, pathogen-associated molecular patterns.

In summary, cell pyroptosis can be triggered by multiple proteins and signaling pathways that are both distinct and interrelated (Fig. 2). Thus, elucidating the molecular mechanisms of cell pyroptosis will facilitate the development of targeted strategies to modulate this form of cell death, thereby protecting the body from pyroptosis-associated damage.

### 3.3 The Association of Pyroptosis With Other Hot Research Topics in MIRI

#### 3.3.1 Cuproptosis 

Cuproptosis is a novel form of regulated cell death. The main mechanism of copper-induced death involves the intracellular accumulation of copper ions, which directly bind to lipoyl moieties within the tricarboxylic acid (TCA) cycle, leading to protein aggregation, dysregulation, TCA cycle disruption, protein toxicity stress, and induction of cell death [60]. Studies have shown that levels of cell death-related genes and proteins increase significantly with elevated copper levels in porcine jejunal epithelial cells treated with excess copper, indicating that copper overload can induce cell death [61]. In addition, copper overload has been shown to mediate macrophage pyroptosis and regulate the inflammatory response by activating the NLRP3 inflammasome. In a mouse model of NLRP3 activation-induced inflammation by intraperitoneal injection of *Escherichia coli*-derived lipopolysaccharide after pretreatment with the copper chelator Trillium tschonoskii Maxim (TTM), a decrease in serum caspase-1-dependent cytokines was observed, whereas caspase-1-independent cytokines were not affected by TTM pretreatment [62]. Recent research has moved beyond conceptual links to define specific cuprototic pathways active in the heart. Indeed, a pivotal 2025 study revealed that in MIRI, the upregulated protein proprotein convertase subtilisin/kexin type 9 (PCSK9) directly binds to lipoyl synthase (LIAS), a key mitochondrial enzyme, and triggers cuproptosis in cardiomyocytes. Therapeutic inhibition of PCSK9 with evolocumab disrupted this interaction, alleviated cardiac damage, and improved function in mice, identifying a novel cardioprotective target [63]. These studies suggest a close connection between cuproptosis and pyroptosis.

#### 3.3.2 N6-Methyladenosine

N6-methyladenosine (m6A) is the most prevalent RNA modification, present in the mRNA of most eukaryotes and some viruses. Meanwhile, m6A methylation is the process through which RNA molecules selectively add a methyl group to specific adenine residues via the RNA methyltransferase complex [64]. Recent reports have revealed the involvement of cell death in diabetic retinopathy and identified a close association with m6A methylation mediated by methyltransferase-like protein 3 (METTL3) [65]. Moreover, METTL3 regulates the occurrence of pyroptosis through m6A modification of MALAT1 [66]. Diao *et al*. [67] discovered the regulatory role of the phosphatase and tensin homolog (PTEN) in cell death. The study indicated that *PTEN* mRNA with m6A sites can inhibit NLRP3 inflammasome activation and the expression of cell death-related proteins through the phosphatidylinositol 3-kinase (PI3K)/Akt/GSK-3β signaling axis, thereby significantly preventing the secretion of proinflammatory cytokines IL-18 and IL-1β. Another fundamental study indicated that the interferon-induced transcription factor IRF-1 promotes macrophage pyroptosis by inhibiting the expression of *hsa_circ_0029589* and upregulating m6A and METTL3 expression, indicating that m6A modification plays a crucial role in IRF-1-induced macrophage pyroptosis [68]. A key study found that the m6A “reader” protein YTHDF2 recognizes increased m6A modification on the mRNA of *MG53* (*TRIM72*), a protein vital for sarcolemmal membrane repair. YTHDF2-mediated degradation of *MG53* mRNA compromises the intrinsic repair capacity of cardiomyocytes, thereby exacerbating cell death following hypoxia/reoxygenation and ischemia/reperfusion [63]. Furthermore, in the coronary microvasculature, downregulation of the methyltransferase METTL14 in endothelial cells destabilizes *USP48* mRNA (a deubiquitinase), leading to mitochondrial dysfunction and increased oxidative stress and, consequently, aggravating microvascular injury during MI [69]. These studies delineate how m6A modification, through writers (*e*.*g*., METTL14) and readers (*e*.*g*., YTHDF2), post-transcriptionally governs the expression of proteins critical for cardiomyocyte survival and endothelial function in MIRI. Therefore, these studies highlight the significant association between m6A methylation and pyroptosis, indicating that m6A modification influences the occurrence of cell death by modulating distinct molecular pathways.

#### 3.3.3 Ubiquitination 

Ubiquitination is a process in which a class of low-molecular-weight proteins undergoes specific enzymatic reactions to categorize intracellular proteins, select target substrates, and mediate specific modifications of the target proteins [70]. These key enzymes include ubiquitin-activating enzymes, conjugating enzymes, ligases, and degrading enzymes. Ubiquitination plays a crucial role in the localization, metabolism, function, regulation, and degradation of proteins. Simultaneously, ubiquitination also participates in the regulation of nearly all vital cellular processes, including the cell cycle, proliferation, pyroptosis, DNA repair, and inflammatory immunity [71]. NLRP3, a member of the NOD-like receptor family, can broadly detect various stimuli, including pathogenic microorganisms, bacterial toxins, and inflammatory signals [72]. Activation of NLRP3 is a prerequisite for the assembly of the NLRP3 inflammasome and pyroptosis [73]. Increasing evidence suggests that the ubiquitin–proteasome system influences the assembly and activation of the NLRP3 inflammasome, and that deubiquitination of NLRP3 is crucial for activating the NLRP3 inflammasome [74]. A seminal study showed that ubiquitination of liver kinase B1 (LKB1) significantly inhibited the NLRP3 inflammasome response via the LKB1/AMPK pathway, ultimately reducing pyroptosis [75]. Additionally, ROS generation was identified as a critical link in regulating NLRP3 inflammasome activation. The accumulation of cytoplasmic ROS disrupts the interaction between NLRP3 and ubiquitin. Importantly, when cytoplasmic ROS are eliminated using N-acetylcysteine, NLRP3 reverts to a polyubiquitinated state, leading to significant downregulation of NLRP3 inflammasome-related proteins and a significant decrease in pyroptosis [76]. A novel mechanism involves the E3 ligase Listerin, which catalyzes K63-linked polyubiquitination of the cholesterol transporter ABCA1. This non-degradative ubiquitination stabilizes ABCA1, promoting cholesterol efflux from macrophages and attenuating atherosclerosis—a primary cause of ischemic events [77]. Collectively, these studies indicate that ubiquitination significantly influences the incidence of pyroptosis.

#### 3.3.4 miRNA

MicroRNAs (miRNAs) are a class of small single-stranded RNAs closely associated with the regulation of gene expression [78]. Notably, miRNAs regulate pyroptosis by binding to the 3^′^ untranslated regions of various pyroptosis-related protein mRNAs, thereby inhibiting their translation or inducing their degradation [79]. Studies have shown a significant increase in the expression of *miRNA-30d* in streptozotocin-induced type 2 diabetes (T2DM) rats and cardiomyocytes treated with high glucose. The upregulation of *miRNA-30d* promotes caspase-1 expression and the release of the proinflammatory cytokines IL-1β and IL-18, resulting in structural and functional changes in T2DM rats, including myocardial interstitial fibrosis and a significant decrease in ejection fraction. Conversely, knockout of *miRNA-30d* attenuates the expression of pyroptosis-related proteins, thereby alleviating cardiomyocyte damage [80]. Overexpression of *miRNA-9* in human cardiomyocytes directly targets ELAV-like RNA-binding protein 1, downregulating caspase-1 and IL-1β, inhibiting cardiomyocyte pyroptosis, and reducing the incidence of heart failure [81]. Additionally, overexpression of *miRNA-141-3p* reduces the proportion of pyroptotic cells and the expression levels of pyroptosis-related proteins in high-glucose-treated H9C2 cardiomyocytes. Similarly, overexpression of *miRNA-214-3p* significantly decreases the expression levels of NLRP3, caspase-1, and IL-1β in T2DM patients and in AC16 cardiomyocytes cultured under high-glucose conditions, thereby inhibiting pyroptosis [82]. These studies collectively demonstrate that miRNAs regulate pyroptosis.

### 3.4 Drugs Targeting Pyroptosis in MIRI

Recent research suggests that several pyroptosis inhibitors can suppress cardiac inflammation, improve MIRI, enhance cardiac function, and reduce myocardial infarction. As further investigations advance, drugs targeting pathways involving NLRP3, caspase-1, GSDMD, IL-1β, and IL-18 are being identified to modulate pyroptosis and treat MIRI.

In an ischemia-reperfusion model of Sprague–Dawley rats, Peng *et al*. [83] discovered that the ethyl acetate extract of *C. ramulus* and the associated bioactive component cinnamic acid can attenuate MIRI by inhibiting NLRP3 inflammasome and cell pyroptosis. Cinnamaldehyde, one of the main active compounds in cinnamon, was shown in a rat ischemia-reperfusion model to counteract MIRI by inhibiting NLRP3 inflammasome activation and GSDMD-mediated myocardial pyroptosis [84]. Aesculin, a hydroxycoumarin glycoside with various biological properties, was found by Xu *et al*. [85] to protect cardiomyocytes from MIRI by inhibiting NLRP3 inflammasome-mediated pyroptosis in a rat ischemia-reperfusion model. Cathepsin B (CTSB) plays a crucial role in regulating cell death, inflammatory responses, and angiogenesis. In a rat ischemia-reperfusion model, Ilexsaponin I (ISI), a triterpene saponin extracted from holly, significantly inhibited CTSB-triggered NLRP3 inflammasome activation and reduced the maturation of IL-1β and IL-18, mainly due to ISI promoting the formation of CTSB/HSP70 complexes, disrupting the CTSB/NLRP3 complex, inactivating the NLRP3 inflammasome, and ultimately inhibiting the occurrence of pyroptosis, thus alleviating MIRI [86]. In another experiment, sevoflurane was found to mitigate MIRI by inhibiting pyroptosis through the circPAN3/miR-29b-3p/SDF4 axis [87]. Similarly, in a rat ischemia-reperfusion model, piperazine ferulate was also found to control pyroptosis by regulating NLRP3 inflammasome formation, ultimately reducing MIRI [88]. In a rat ischemia-reperfusion model, Wang *et al*. [9] demonstrated that dexmedetomidine (Dex) could alleviate MIRI by regulating the miR-665/MEF2D/Nrf2 axis to inhibit pyroptosis. Additionally, Dex was found to suppress pyroptosis by downregulating miR-29b*,* thereby activating the FoxO3a/ARC axis and further reducing MIRI [10]. Extracellular vesicles (EVs) derived from hypoxia-preconditioned mesenchymal stem cells (MSCs) also provide robust cardiac protection against MIRI. EVs isolated from hypoxia-preconditioned or normoxia-treated adipose tissue-derived MSCs (ADSCs) in mice were evaluated for their ability to promote survival of mouse cardiomyocytes *in vivo* following MIRI and *in vitro* after hypoxia/reoxygenation (H/R). In mice subjected to MIRI, injection of hypoxia-preconditioned ADSC-EVs significantly reduced pyroptosis and infarct area compared with normoxia-treated ADSC-EVs (NC-EVs) [89]. Insulin protects against MIRI by regulating PDHA1 dephosphorylation, a mechanism that reduces NLRP3-induced pyroptosis to alleviate MIRI [90]. Collectively, these studies indicate that pyroptosis can be inhibited through various pathways, ultimately alleviating MIRI (Table 2, Ref. [9, 83, 84, 85, 86, 87, 88, 89, 90]).

**Table 2.** **Drug studies targeting pyroptosis in myocardial ischemia-reperfusion injury**.

Researcher	Year	Model	Target	Drug
Peng *et al*. [83]	2021	I/R-treated rat	NLRP3, IL-1β, IL-6, TNF-α	Ethyl acetate extract of *Cinnamomi Ramulus*
Luan *et al*. [84]	2022	I/R-treated rat	NLRP3/caspase-1/GSDMD	Cinnamaldehyde
Xu *et al*. [85]	2021	I/R-treated rat, OGD/R-treated NRCMs	NLRP3, Akt/GSK3β/NF-κB	Aesculin
Wu *et al*. [86]	2022	I/R-treated rat, OGD/R-treated H9c2	NLRP3, cathepsin B/HSP70 complex	Ilexsaponin I
An *et al*. [87]	2023	I/R-treated rat, H/R-treated HCMs, 293 T cell lines	Circular RNA PAN3/microRNA-29b-3p/stromal cell-derived factor 4 axis	Sevoflurane
Lei *et al*. [88]	2022	I/R-treated rat	NLRP3	Piperazine ferulate
Wang *et al*. [9]	2023	I/R-treated rat, H/R-treated H9c2	miR-665/MEF2D/Nrf2 axis	Dexmedetomidine
Mao *et al*. [89]	2021	I/R-treated C57, H/R-treated NRCMs	TXNIP	Extracellular vesicles
Pan *et al*. [90]	2023	I/R-treated rat	PDHA1	Insulin

I/R, ischemia-reperfusion; TXNIP, thioredoxin-interacting protein; OGD, oxygen-glucose deprivation; HCMs, neonatal rat cardiomyocytes; GSK, glycogen synthase kinase; MEF2D, myocyte enhancer factor 2d; ARC, apoptosis repressor with caspase recruitment domain; PDHA1, pyruvate dehydrogenase e1 subunit alpha 1.

In the context of MIRI, pyroptosis is a significant mechanism underlying myocardial damage. Therefore, targeting pyroptosis with specific drugs has the potential to reduce cardiomyocyte death and protect cardiac tissue from damage. Overall, modulation of pyroptosis with targeted agents represents a promising strategy for treating MIRI, offering additional therapeutic options in clinical practice and enhancing treatment efficacy.

## 4. Conclusions and Perspectives 

Pyroptosis, a recently redefined form of regulated cell death, has been confirmed by numerous studies to play a crucial role in MIRI. Despite being a well-studied aspect within MIRI, several pressing issues remain. Firstly, during MIRI, pyroptosis coexists with other forms of cell death, such as apoptosis, necrosis, and autophagy. Therefore, investigating the relationships between cellular pyroptosis and these other cell death modalities will help elucidate the mechanisms of ferroptosis in the MIRI process and enable more effective inhibition of MIRI-triggered cardiomyocyte death. Secondly, drug development targeting cellular pyroptosis in MIRI remains largely confined to basic research. Although these substances are effective in inhibiting myocardial pyroptosis post-MIRI in animal studies, significant progress in clinical practice remains to be achieved. Moreover, research on the impact of the drugs currently approved for clinical use on pyroptosis remains limited. Thus, pharmaceutical treatment targeting pyroptosis in MIRI requires further reinforcement in clinical practice. Finally, MIRI is a dynamic process, and pyroptosis changes with the development of ischemia and reperfusion. Hence, investigating these dynamic changes will help more accurately reveal the pathophysiological mechanisms of MIRI.

In summary, pyroptosis in MIRI holds significant research value. In-depth exploration of the regulatory mechanisms underlying pyroptosis during the MIRI process will yield new perspectives and strategies for preventing and treating MIRI-related injuries.
